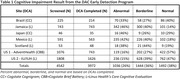# Implementing Digital Cognitive Assessments to Detect Cognitive Impairment: Results from the Davos Alzheimer’s Collaborative Early Detection Program

**DOI:** 10.1002/alz.087937

**Published:** 2025-01-09

**Authors:** Tim MacLeod, James F. Murray, Otelo Corrêa dos Santos Filho, Marcilea Dias de Sá Paiva Lima, Ishtar Govia, Janelle Robinson, Hisatomo Kowa, Kohei Morimoto, Mariana López‐Ortega, Alison McKean, Craig Ritchie, Magda R. Baksh, Steven R. Smith, Deanna R Willis, Jared R. Brosch, Seamus Small, Tammy Martin, Katherine J. Selzler

**Affiliations:** ^1^ Davos Alzheimer’s Collaborative, Wayne, PA USA; ^2^ Human Aging Center, State University of Rio de Janeiro, Rio de Janeiro, Rio de Janeiro Brazil; ^3^ Municipal Health Department of Volta Redonda, Rio de Janeiro Brazil; ^4^ Jamaica Mental Health Advocacy Network, Kingston Jamaica; ^5^ CUNY Graduate Center, New York, NY USA; ^6^ Kobe University, Kobe Japan; ^7^ Instituto Nacional de Geriatría (National Institute of Geriatric), Mexico City, EM Mexico; ^8^ Brain Health Scotland, Edinburgh United Kingdom; ^9^ Scottish Brain Sciences, Edinburgh United Kingdom; ^10^ AdventHealth, Orlando, FL USA; ^11^ Indiana University School of Medicine, Indianapolis, IN USA; ^12^ Linus Health, Boston, MA USA; ^13^ Cogstate, New Haven, CT USA

## Abstract

**Background:**

Cognitive impairment is frequently undetected or undiagnosed in the early stages. To increase the rates of detecting cognitive impairment, the Early Detection program of the Davos Alzheimer’s Collaborative System Preparedness (DAC‐SP) implemented digital cognitive assessments (DCA) in primary care and other non‐specialty settings.

**Methods:**

The DAC‐SP Early Detection program was initiated in 2021 in seven healthcare systems across six countries. Sites were able to choose from several DCAs, and clinicians were provided training, including recognizing signs and symptoms of cognitive decline, and provided with post‐diagnostic support. Patients were eligible for a DCA if they were over 60 years of age, able to hear and see well enough to complete the assessments, and had no prior diagnosis of dementia. The DCA tools included Linus Health’s Core Cognitive Evaluation, Cogstate Cognigram, and Cogstate Brief Battery.

**Results:**

The DCA results across the seven sites are presented in Table 1. There was notable variability in the number of patients screened across sites, which could be attributed to multiple factors (i.e., number of clinics onboarded/trained, additional testing for language and culture appropriateness of DCA tool prior to deployment, reduction in the number of elderly people visiting clinics during the COVID‐19 pandemic, available time of clinicians, etc.). The rate of cognitive impairment (abnormal and borderline) was also numerically higher at sites outside the US, independent of the DCA tool used. However, this study was not designed to evaluate operating characteristics of DCA tools, so further research is needed. Approximately 60% of the patients in the DAC‐SP Early Detection program tested abnormal or borderline for cognitive issues, suggesting the need for additional clinical assessment and follow‐up.

**Conclusion:**

Findings from the DAC‐SP Early Detection program demonstrated a DCA can be implemented in existing patient care workflows, including primary care settings, and across healthcare systems globally with different resource settings. Adoption of DCAs in clinical practice can help improve the ability to detect symptoms of cognitive impairment and provide much needed earlier screening and care for patients and their families.